# Dangerous snakes, deadly snakes and medically important snakes

**DOI:** 10.1186/1678-9199-19-26

**Published:** 2013-10-07

**Authors:** Anjana Silva

**Affiliations:** 1 Department of Parasitology, Faculty of Medicine and Allied Sciences, Rajarata University of Sri Lanka, Anuradhapura 50008, Sri Lanka

**Keywords:** Snakes, Snake bites, Snake venoms, Snake envenomation

## Abstract

This correspondence argues that the dangerousness of a venomous snake species is not solely determined by the venom characteristics or the lethality of the snake, and recognizes that medical importance comprises a key variable as well. The medical importance of a snake is determined by several factors – including frequency of medical attention after a bite, local or systemic envenomation provoked by the bite, fatal bites, long term consequences, availability of antivenom therapy as well as the size of the population at risk – that may vary from one region to another.

## 

Dear Editor of JVATiTD,

The present letter is a response to the text “What is the most dangerous snake?” by McCue MD, published in your journal [1].

Misconceptions are common regarding snakes and snakebites, subjects that have drawn much attention and curiosity among humans across a range of cultures and civilizations. However, such negative beliefs are not limited to the popular science. Misconceptions tend to mask the true picture of snakebites and may result in neglect of known medically important snakes as well as undue recognition of other relevant snake species. Whilst in agreement with McCue’s [1] view on the misapprehension about the most dangerous snake in popular science, I would like to further emphasize the necessity of the recognition of the dangerousness of snakes based on their medical importance.

Death is an extreme outcome, not the sole representation of the harmful effects of snakebite. Therefore, lethality is only one factor that contributes towards the medical importance of a snake species. Furthermore, geographical variations that affect snake venoms, even within the same species, have led to contrastingly different outcomes in patients from different regions bitten by the same snake species [2]. Moreover, the effects of snakebites on humans are not solely dependent on venom characteristics, but, more importantly, may be associated with other factors including age and general health of the victim, availability of medical care, and early administration of effective antivenom [3]. In addition, the effects of snakebites in a community are further determined by the exposure level of the population at risk, frequency of bites that require medical attention, possibility of causing life-threatening envenomations and long-term disabilities associated with the bites. This means that the dangerousness of a venomous snake species, in terms of the medical importance, could be different among geographical regions or human populations.

Saw scaled viper (*Echis carinatus*) is considered a dangerous snake where it is found and causes numerous envenomations in South Asia [4]. In Sri Lanka, saw scaled vipers are responsible for 1 to 2% of the 37,000 snakebites reported to hospitals annually [5]. These snakes are restricted to coastal arid areas of Northern Province of Sri Lanka, where human population density is low compared to other parts of the country. Systemic envenomation is common among victims of these snakes in the country [6]. Comparatively, Merrem’s hump-nosed pit vipers (*Hypnale hypnale*) provoke 35 to 45% of the snakebites annually reported in Sri Lanka, and are distributed throughout the island occupying a wide range of anthropogenic habitats [5,7]. Few fatal *H. hypnale* bites have been reported and systemic envenomation by these snakes is also less common. However, these snakes commonly cause severe local envenomation that requires medical attention [8]. The only available antivenom in Sri Lanka, the Indian polyvalent antivenom, covers saw scaled viper bites but does not work for *H. hypnale* bites. Based on these variables, it is clear that the most dangerous snake between the two species in terms of medical importance in Sri Lanka is Merrem’s hump-nosed pit viper.

Therefore, medical importance based on public health factors must be taken into account in the discussion on dangerousness of a snake species. Leaving aside the argument on the most dangerous snake in the world, I wish to emphasize that the dangerousness of a snake species, based on its medical importance, is particular to different human populations in distinct geographical regions.

## Competing interests

The author declares that he is no competing interests.

## References

[B1] McCue MD What is the most dangerous snake? J Venom Anim Toxins incl Trop Dis 2013 19 1 19 10.1186/1678-9199-19-19 23981479 PMC3766188

[B2] Thorpe RS Pook CE Malhotra A Phylogeography of the Russell’s viper (*Daboia russelii*) complex in relation to variation in the colour pattern and symptoms of envenoming Herpetol J 2007 17 4 209 218

[B3] Warrell DA Snake bite Lancet 2010 375 9708 77 88 10.1016/S0140-6736(09)61754-2 20109866

[B4] Alirol E Sharma SK Bawaskar HS Kuch U Chappuis F Snake bite in South Asia: a review PLoS Negl Trop Dis 2010 4 1 e603 10.1371/journal.pntd.0000603 20126271 PMC2811174

[B5] Kasturiratne A Pathmeswaran A Fonseka MM Lalloo DG Brooker S de Silva HJ Estimates of disease burden due to land-snake bite in Sri Lankan hospitals Southeast Asian J Trop Med Public Health 2005 36 3 733 740 16124448

[B6] Kularatne SA Sivansuthan S Medagedara SC Maduwage K de Silva A Revisiting saw-scaled viper (*Echis carinatus*) bites in the Jaffna Peninsula of Sri Lanka: distribution, epidemiology and clinical manifestations T Roy Soc Trop Med H 2011 105 10 591 597 10.1016/j.trstmh.2011.07.010 21868049

[B7] Maduwage K Silva A Manamendra-Arachchi K Pethiyagoda R A taxonomic revision of the South Asian hump-nosed pit viper genus *Hypnale* (Fitzinger) Zootaxa 2009 2232 1 28

[B8] Maduwage K Isbister GK Silva A Bowatta S Mendis S Gawarammana I Epidemiology and clinical effects of hump-nosed pit viper (Genus: *Hypnale*) envenoming in Sri Lanka Toxicon 2013 61 11 15 23127899 10.1016/j.toxicon.2012.10.013

